# Arginine‐ but not alanine‐rich carboxy‐termini trigger nuclear translocation of mutant keratin 10 in ichthyosis with confetti

**DOI:** 10.1111/jcmm.14727

**Published:** 2019-10-22

**Authors:** Patricia Renz, Elias Imahorn, Iris Spoerri, Magomet Aushev, Oliver P. March, Hedwig Wariwoda, Sarah Von Arb, Andreas Volz, Peter H. Itin, Julia Reichelt, Bettina Burger

**Affiliations:** ^1^ Department of Biomedicine University Hospital Basel and University of Basel Basel Switzerland; ^2^ Wellcome Centre for Mitochondrial Research Institute of Genetic Medicine Newcastle upon Tyne UK; ^3^ Department of Dermatology EB House Austria University Hospital of the Paracelsus Medical University Salzburg Austria; ^4^ Dermatology University Hospital Basel Basel Switzerland

**Keywords:** alanine‐rich C‐terminus, arginine‐rich C‐terminus, carboxy terminus, ichthyosis with confetti, keratin 10, KRT10, nuclear localization

## Abstract

Ichthyosis with confetti (IWC) is a genodermatosis associated with dominant‐negative variants in *keratin 10* (*KRT10*) or *keratin 1* (*KRT1*). These frameshift variants result in extended aberrant proteins, localized to the nucleus rather than the cytoplasm. This mislocalization is thought to occur as a result of the altered carboxy (C)‐terminus, from poly‐glycine to either a poly‐arginine or ‐alanine tail. Previous studies on the type of C‐terminus and subcellular localization of the respective mutant protein are divergent. In order to fully elucidate the pathomechanism of IWC, a greater understanding is critical. This study aimed to establish the consequences for localization and intermediate filament formation of altered keratin 10 (K10) C‐termini. To achieve this, plasmids expressing distinct *KRT10* variants were generated. Sequences encoded all possible reading frames of the K10 C‐terminus as well as a nonsense variant. A keratinocyte line was transfected with these plasmids. Additionally, gene editing was utilized to introduce frameshift variants in exon 6 and exon 7 at the endogenous *KRT10* locus. Cellular localization of aberrant K10 was observed via immunofluorescence using various antibodies. In each setting, immunofluorescence analysis demonstrated aberrant nuclear localization of K10 featuring an arginine‐rich C‐terminus. However, this was not observed with K10 featuring an alanine‐rich C‐terminus. Instead, the protein displayed cytoplasmic localization, consistent with wild‐type and truncated forms of K10. This study demonstrates that, of the various 3′ frameshift variants of *KRT10*, exclusively arginine‐rich C‐termini lead to nuclear localization of K10.

## 1 INTRODUCTION

Ichthyosis with confetti (IWC), also termed congenital reticular ichthyosiform erythroderma (CRIE) or ichthyosis variegata (OMIM #609165), is a keratinopathy associated with dominant‐negative frameshift variants in *keratin 10* (*KRT10*) and *keratin 1* (*KRT1*).^1^, ^2^ Skin of affected patients demonstrates generalized erythroderma, persisting throughout life. The disease is characterized by the appearance of numerous patches of pale, healthy‐appearing skin in most patients during childhood. These increase in size and number with age.^3^, ^4^, ^5^ Currently, 57 patients from 40 families (49% male, 51% female) have been reported (Table 1). Of these, 50 were sequenced. The majority of these individuals (69%) display a heterozygous variant in *KRT10* (familial 82%), whereas 31% carry a heterozygous variant in *KRT1* (familial 18%).

**Table 1:** Overview of reported patients with IWC

No of patients	No of families	Sex		Clinically described	Without spots	Affected gene		Type of variant	Reference
		Male	Female			*KRT1*	*KRT10*		
1*	1	1		1				Unknown	4
1*	1		1	1				Unknown	30
1*	1	1		1				Unknown	31
1*	1		1	1				Unknown	32
1*	1		1	1				Unknown	33
1*	1	1		1				Unknown	34
**6**	**6**	**3**	**3**	**6**					
1	1		1	1			c.1370G>T†	Exon 6	1, 35
1	1	1		1			c.1373+1delG††	Donor splice site	10
3	1	2	1				c.1373+1G>A	Donor splice site	1
3	1	1	2	3	2		c.1373+1G>C	Donor splice site	14
1	1	1		1			c.1373+2T>C	Donor splice site	14
1	1		1				c.1374‐2delA	Acceptor splice site	1
1	1	1		1	1		c.1374‐2A>C	Acceptor splice site	36
2	2	2		1			c.1374‐2A>G	Acceptor splice site	1, 37
4	3	1	2	2			c.1374‐1G>A	Acceptor splice site	1, 5, 38
5	5	3	3	6	1		c.1374‐1G>C	Acceptor splice site	5, 14, 39, 40, 41
1	1		1	1			c.1383_1414del	Exon 7	39
1	1		1	1			c.1411_1412insA	Exon 7	14
1	1	1					c.1449_1450insC	Exon 7	1
1	1	1		1			c.1452_1464delinsAG	Exon 7	42
1	1		1	1			c.1506_1507del	Exon 7	4, 5, 43, 44
2	2		2	2			c.1544dupG‡	Exon 7	11, 14
1	1		1	1			c.1546_1551delinsT	Exon 7	3, 5, 45
1	1		1	1			c.1557_1558delCG	Exon 7	5
2	1	1	1				c.1560_1561delGC	Exon 7	1
2	1		2	1			c.1573dupA	Exon 7	5
**35 (69%)**	**28 (82%)**	**15**	**20**	**25**	**4**		**20**	**43% exonic, 57% splice site (families)**	
6	1	2	4	5	3^a^	c.591+329_1129‐37del		Exon 9	46
1	1	1		1	1^b^	c.1756_1757insG^†††^		Exon 9	47
1	1	1		1	1^b^	c.1752dupT		Exon 9	14
3	1	1	2	3	1	c.1758_1759insT		Exon 9	48
1	1	1		1	1^c^	c.1860_1861insT		Exon 9	49
4	1	4		4		c.1865_1866insG		Exon 9	2
**16 (31%)**	**6 (18%)**	**10**	**6**	**15**	**7**	**6**		**100% exonic**	

Note

All IWC patients carry heterozygous variants in KRT10 (NM_000421) or KRT1 (NM_006121). Two variants, originally described as a poly‐alanine reading frame, have been redefined according to HGVS nomenclature (indicated by ^††^ and ^‡^). Six patients, which have not been sequenced but displayed typical IWC clinical features, including pale spots, are also listed (indicated by *). Data include eleven patients from different families who have not currently developed pale spots but carry a poly‐arginine tail‐causing frameshift variant. Of these, three siblings in a familial IWC resembled epidermolytic ichthyosis (EI) (indicated by ^a^). Three sporadic cases were clinically described as EI (indicated by ^b^) or ichthyosis hystrix Curth‐Macklin (IHCM) (indicated by ^c^).

a Resembled epidermolytic ichthyosis (EI).

b Described as epidermolytic hyperkeratosis, renamed as epidermolytic ichthyosis (EI) (OMIM #113800).

c Described as ichthyosis hystrix Curth‐Macklin (OMIM #146590).

^†^ Originally described as c.1369G>T; results in a new donor splice site.

^††^ Originally described as c.1373delG (alanine‐rich C‐terminus).

^†††^ Originally described as c.1751insG.

^‡^ One of them initially described as c.1544delG (alanine‐rich C‐terminus).

Keratins comprise the components of the epithelial cytoskeleton. Acidic (type I) and basic (type II) keratins heterodimerize and subsequently assemble to form intermediate filaments (IFs).^6^ Epidermal keratinocytes specifically co‐express keratins as pairs in a differentiation‐dependent manner. Keratinocytes of the proliferative basal layer express keratin (K) 5 and K14. The differentiating suprabasal epidermal layers are associated with the down‐regulation of K5/K14 and expression of thicker K1/K10 filament bundles.^7^

All currently described IWC‐associated variants are deletions, insertions or duplications located within the helix‐termination‐motif or in the V2 domain of either K10 or K1 (Table 1). Each results in frameshifts, leading to the translation of aberrant‐truncated protein tails. Nonsense variants in the V2 domain of either K10 or K1 have not been reported. Variants leading to −2/+1 frameshifts within regions encoding the V2 domain are predicted to alter the downstream amino acid code from native glycine‐ to arginine‐rich sequences. Arginine‐rich sequences frequently function as nuclear localization signals.^8^, ^9^ Therefore, aberrant arginine‐rich K10 or K1 tails might be responsible for the pathogenic nuclear localization of K10 or K1, characteristic of IWC.

Two studies suggested that alanine‐rich K10 tails might also lead to nuclear K10 localization.^10^, ^11^ In contrast to arginine, alanine‐rich sequences have not been described to function as nuclear localization signal.^12^ Lim et al,^10^ suggested that a *KRT10* frameshift variant at the exon 6/intron 6 boundary resulted in an alanine‐rich tail. However, the original c.1373delG nomenclature for this variant should rather be annotated as c.1373+1delG, according to the sequence variant nomenclature HGVS,^13^ indicating a splice site variant. Variants of this nature often lead to the expression of multiple splicing products. Full examination of the expression products of this variant is therefore justified. The presence of additional K10 splicing products, featuring arginine‐rich tails, may explain the nuclear K10 localization displayed in the epidermis of this patient. Hotz et al^11^ also suggested that an IWC‐associated variant (c.1544delG) resulted in an alanine‐rich K10 tail. However, the variant was later on corrected to c.1544dupG and has been suggested to result in an arginine‐rich K10 tail.^14^

In support of a non‐nuclear localizing signal of alanine‐rich keratin tails, frameshift variants in *KRT1,* leading to alanine‐rich tails, are reported to cause ichthyosis hystrix Curth‐Macklin (IHCM)^15^, ^16^ and striate palmoplantar keratoderma (SPPK).^17^ Keratinocytes of these patients do not display nuclear localization of aberrant K1 products.^16^

The aim of this study was to clarify the effects of mutant K10 tail variants on the pathogenic nuclear localization of the protein. In order to elucidate this, we established two distinct cell culture models. The first model (*transgenic model)* centred on the transient expression of N‐terminal eGFP‐labelled K10 cDNA or gDNA, featuring mutant K10 tail variations, in an immortalized wild‐type human keratinocyte line (NKc21). This model enabled determination of the subcellular localization of K10 translated from IWC‐associated alleles. In vivo, the effects of *KRT10* variants are not observable in undifferentiated keratinocytes. Endogenous regulation of *KRT10* expression ensures equal ratios of mutant and wild‐type K10. Therefore, a context more closely resembling the in vivo situation enabled better analysis of the effect of IWC‐associated alleles. This was achieved through the second model (*endogenous model*), generated via CRISPR/Cas9‐mediated gene editing, which featured these variants at the endogenous *KRT10* locus in NKc21 keratinocytes.

This study elucidates the relationship between K10 C‐termini variants and K10 nuclear localization. The influence of C‐termini variants on keratinocyte differentiation, K10 polymerization with assembly partners and the intracellular localization of these polymers were additionally characterized.

## 2 MATERIAL AND METHODS

### 2.1 Plasmids and transfection

**Figure 1:**
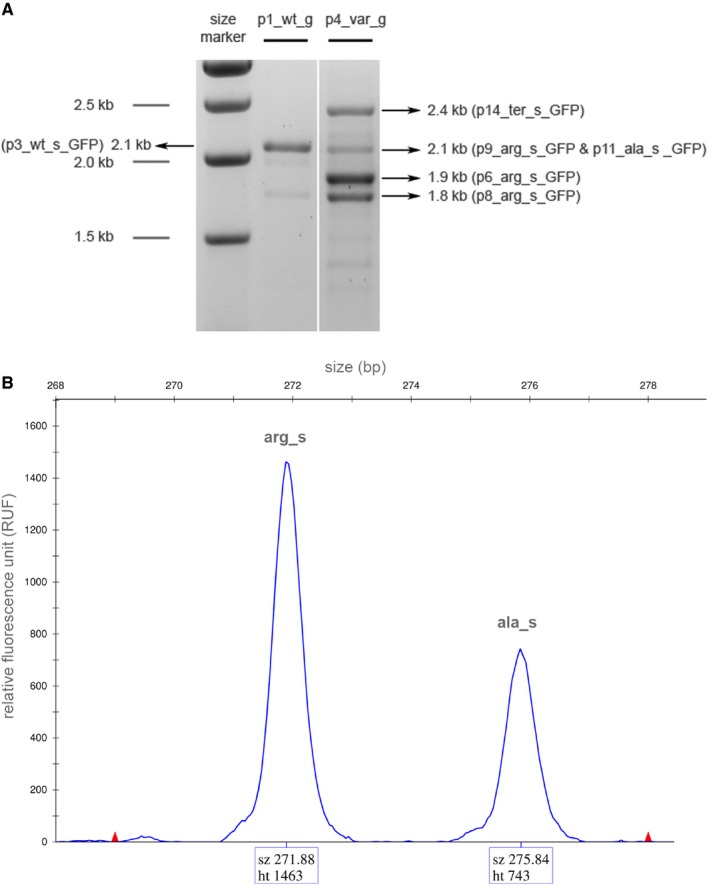
Products amplified from total *KRT10* mRNA of NKc21 cells after transfection with either wild‐type or IWC‐associated *KRT10* gDNA. A. PCR‐amplified cDNA product, following transfection of NKc21 cells with wild‐type plasmid (p1_wt_g_GFP), was at the expected wild‐type size (~2.1 kb). Transfection with IWC‐associated *KRT10* gDNA (p4_var_g_GFP) resulted in four major products, of distinct sizes (~2.4, ~2.1, ~1.9 and ~1.8 kb). Each product was cloned into pEGFP‐C1 for further studies. B. Unsaturated PCR following RT‐PCR of transcripts after transfection with p4_var_g_GFP, using primers localized in exon 6 and 7. These only amplify the 2.1 kb products. Separation of the resulting products (3130xl Genetic Analyzer, Applied Biosystems) showed peaks at 272 bp (arg_s) and 276 bp (ala_s). Measurement of the area under the curve (ht) revealed the quantity of PCR product, almost double the amount of the 4 bp shorter *KRT10_arg_* (arg_s, r.1369_1373del) vs. *KRT10_ala_* (ala_s, r.1373del) PCR product. Red triangles indicate the size marker (GeneScan™ 500 ROX™ (ThermoFisher Scientific Inc))

cDNA‐derived *KRT10* was PCR‐generated from epidermal mRNA isolated from an IWC patient^3^ (p5_arg_c_GFP) or a healthy control (p2_wt_c_GFP). This was cloned into XhoI and HindIII sites of the pEGFP‐C1 vector (Clontech Laboratories Inc Takara). Sequences encoding the alanine‐rich C‐terminus (p10_ala_c_GFP) or a premature stop (p13_ter_c_GFP) were inserted into p2_wt_c_GFP via site‐directed mutagenesis (GenScript^®^). Genomic DNA‐derived *KRT10* from a healthy control was cloned into pUC19 in two steps following amplification of *KRT10* as 3′ and 5′ products. Initially, intron 5 to 3′‐UTR gDNA was inserted into the HincII and HindIII restriction sites of pUC19 (pUC_K10‐ex5‐3′). Subsequently, the 5′ product (mid of exon 1 to exon 4) was introduced into the SacI and HincII restriction sites of pUC_K10‐ex5‐3′. This combined product (pUC‐K10) was inserted into the SacI and HindIII restriction sites of p2_wt_c_GFP, exchanging cDNA between mid of exon 1 to 3′‐UTR for a complete gDNA sequence. This wild‐type gDNA construct (p1_wt_g_GFP) contained a common 12 bp deletion in exon 7 (rs778613907)^5^ enabling it to be distinguished from endogenous *KRT10* of NKc21. The IWC100 variant^10^ (c.1373+1delG) (p4_var_g_GFP) was inserted via site‐directed mutagenesis (GenScript^®^). Subconfluent NKc21 keratinocytes were transfected with p1_wt_g_GFP or p4_var_g_GFP to receive the differentially spliced *KRT10* mRNA. After full‐length PCR and separation on 1% agarose gel, they were cloned into pEGFP‐C1 using XhoI and HindIII restriction sites (Figure 1A). To distinguish the *KRT10_wt_*, *KRT10_arg_* and *KRT10_ala_* products following combined transfection in cell imaging analyses, eGFP in p6_arg_s_GFP and p11_ala_s_GFP was replaced with mCherry. Constructs are summarized in Table S1 and Figure S1. NKc21 keratinocytes were transfected with Xfect transfection reagent (Takara Bio Europe) as previously described.^18^ Transfected cells were seeded on coverslips 48 hours post‐treatment and subsequently fixed 24 hours later with 4% formaldehyde. Transfection rates were approximately 30%.

### 2.2 Cell culture

Keratinocyte line NKc21 (kindly provided by Hans Törmä)^19^ was grown in CnT‐PR (CELLnTEC). Cells were cultured in a humid atmosphere at 37°C and 5% CO_2_. Integrity of cells and chromosomes was validated by SNP array (Illumina, HumanOmniExpressExome‐8v1‐3).

Mouse 3T3‐J2 fibroblasts (ATCC) were grown in DMEM (Lonza) supplemented with antibiotic‐antimycotic and 10% foetal calf serum (ThermoFisher Scientific Inc). Feeder cells were generated from this cell line following mitomycin C (StressMarq Biosciences) treatment (4 mg/mL for 2 hours). These were subsequently used in keratinocyte single‐cell cloning.

### 2.3 CRISPR‐Cas9‐mediated gene editing and single‐cell cloning

Plasmid pSpCas9(BB)‐2A‐GFP (PX458) (kindly gifted from Feng Zhang, Addgene plasmid #48138) was used for generation of GFP‐labelled Cas9 vectors.^20^ In brief, the sgRNA oligos were ligated into pSpCas9(BB)‐2A‐GFP after digestion with restriction enzyme BbsI. These resulted vectors carried either guide RNA cr26 (GGCGGCGGAAGTTTCGGCGG, *KRT10,* exon 7) or cr33 (GCCTGCTAGAAGGAGAGGGA *KRT10,* exon 6). Plasmids were transfected with Xfect polymer (Takara Bio) as previously described.^18^ Briefly, 70 000 keratinocytes per well were seeded in 6‐well plates. One day later, a mixture of 660 ng of CRISPR/Cas9 plasmid, 0.2 µL Xfect polymer and 100 µL Xfect buffer was incubated at room temperature for 10 minutes and added to the keratinocytes. After 4 hours at 37°C, the medium was exchanged. Individual GFP‐fluorescent cells were FACSorted 72 hours later on an Aria II cell sorter (BD Biosciences) using 100 µm nozzle and 15 psi pressure. They were co‐cultivated with mouse 3T3‐J2 feeder fibroblasts (ATCC)^21^ previously arrested with 4 g L^−1^ mitomycin C (StressMarq Biosciences) in 96‐well plates. Clones were subsequently expanded. Corresponding sequence data on *KRT10* are summarized in Table S2.

### 2.4 Keratinocyte differentiation and epidermal equivalent formation

Since *KRT10* is only expressed in differentiated cells, differentiation was induced in confluent cultures in the absence of growth factors and at high calcium concentration (1.2 mM). For mRNA isolation, keratinocytes were cultured in CnT‐PR medium (CELLnTEC). One day before expected confluency, medium was exchanged to CnT‐PR‐D (CELLnTEC). At confluency, CaCl_2_ (Vitaris) was added to a final concentration of 1.2 mM. RNA was isolated 72 hours after addition of calcium.

For cellular localization of K10, clonal keratinocyte cultures were seeded onto 0.4 µm PCF Millicell cell culture inserts (Merck Millipore^®^) and cultivated in CnT‐PR medium. After 3 days, cells reached confluency and the medium was exchanged with CnT‐PR‐3D (CELLnTEC to promote differentiation. The following day, medium inside the insert was removed. Medium outside the insert was changed three times a week. Two weeks after the start of differentiation, epidermal models were formalin‐fixed and paraffin‐embedded (FFPE) for subsequent haematoxylin and eosin staining. Each frameshift mutation was analysed with two distinct clones. For K10_arg_ six differentiated clones (2x e3_arg_26‐1, 4x e4_arg_26‐1) and for K10_ala_ nine differentiated clones (6x e5_ala_26‐1, 3x e6_ala_26‐1) were examined.

For quantitation of epidermal thickness, the thickness of keratinocyte layers and insert membrane was measured on haematoxylin‐ and eosin‐stained epidermal equivalents. For K10_arg_ six differentiated clones (2x e3_arg_26‐1, 4x e4_arg_26‐1) and for K10_ala_ seven differentiated clones (4x e5_ala_26‐1, 3x e6_ala_26‐1) were examined. ANOVA tests were used for statistical analysis.

### 2.5 Sequencing of gDNA and RNA

Genomic DNA and mRNA were isolated from confluent keratinocyte cultures 48 hours post‐transfection according to the manufacturer's protocol (NucleoSpin^®^ Tissue and NucleoSpin^®^ RNA XS, Macherey‐Nagel). cDNA was synthesized (Verso cDNA Synthesis kit, ThermoFisher Scientific Inc) and amplified by PCR with *KRT10*‐specific primers (Taq DNA or HotStar HiFidelity polymerase, Qiagen) (Table S3). Sanger sequencing was performed by an external vendor (Microsynth AG).

### 2.6 Ratio of mRNA p9_arg_s_GFP and p11_ala_s_GFP

AB 3130 Genetic Analyzer was used to determine the ratio of *KRT10_arg_* (p9_arg_s_GFP) and *KRT10_ala_* (p11_ala_s_GFP) mRNA within PCR products (Figure 1) by fragment analysis as previously described.^22^ In brief, PCR was performed on cDNA following p4_var_g_GFP transfection with one FAM‐labelled primer. This was stopped after 30 cycles to stay in the log phase of amplification. GeneScan™ 500 ROX™ (ThermoFisher Scientific Inc) was used as size standard. Quantitative assessment of PCR product was achieved following calculation of areas under the curve using GeneMapper.

### 2.7 Immunofluorescence staining

Immunofluorescence staining was performed as previously described both on keratinocytes containing the respective plasmid grown on coverslips and on formalin‐fixed and paraffin‐embedded (FFPE‐slides) keratinocytes of the epidermal models.^3^

In brief, samples were blocked in donkey serum and triton‐X‐100 in TBS for 1 hour and incubated with primary antibodies either at RT for 1 hour (coverslips) or at 4°C overnight (slides). After washing, secondary antibody and DAPI for nuclear staining (Sigma‐Aldrich) were incubated at RT for 1 hour. After additional washing steps, samples were mounted with ProLong™ Diamond Antifade Mountant (ThermoFisher Scientific Inc). The following primary antibodies were used: mouse monoclonal antibodies to the N‐terminus of K10 DE‐K10 (Abcam) and LH2 (kindly gifted by Irene Leigh, Dundee, UK, and Andrew South, Philadelphia, USA), rabbit monoclonal antibodies to the carboxy terminus of K10 SP99 and EP1607IHCY (both Abcam) (Table S4). Additional primary antibodies were goat polyclonal to GFP (Abcam), rabbit polyclonal to K5 (Biolegend) and monoclonal to K14 (EP1612Y, Abcam) as well as polyclonal rabbit to lamin B1 and fibrillarin (both Abcam). Conjugated secondary donkey antibodies originated from Jackson ImmunoResearch and ThermoFisher Scientific Inc Fluorescent signals were imaged under a Zeiss confocal microscope LSM710 with a Zeiss 40x/1.3 and 63x/1.4 objective.

## 3 RESULTS

### 3.1 IWC‐associated KRT10 variants result in an arginine‐rich C‐terminus

NKc21 keratinocytes were transfected with *KRT10* gDNA constructs, featuring either wild‐type (p1_wt_g_GFP) or a previously described IWC‐associated splice site variant (p4_var_g_GFP)^10^ to investigate the presence and composition of potential additional splice products (Table S1). The wild‐type construct resulted in the transcription of one main mRNA product of the expected size (2.1 kb) (wt_s). In contrast, the construct encoding the IWC‐causing sequence resulted in four major mRNA products, of differing sizes (Figure 1A). Each mRNA was distinct (Table S1, Figure S1). Subcloning and sequencing of these products demonstrated deletions of complete exons (r.1156_1373del; subcloned in p6_arg_s_GFP) (r.1030_1373del; subcloned in p8_arg_s_GFP), both would lead to the expression of K10_arg_ (p.Lys386PhefsTer118 and p.Ser344PhefsTer118). The third product (subcloned in p14_ter_s_GFP) contained the complete intron 6, resulting in a shifted reading frame with a stop codon after 4 bp of intron 6. The fourth product contained two sequences, with the majority of products displaying a 5 bp deletion of the 3′‐terminus of exon 6 (r.1369_1373del, p.Gly457PhefsTer118; subcloned in p9_arg_s_GFP) leading to K10_arg_ expression. A minority of products displayed a deletion of the last nucleotide of exon 6 (r.1373del; subcloned in p11_ala_s_GFP), encoding K10_ala_ (p.Ser458IlefsTer157). Quantitative assessment of an unsaturated PCR enabled relative PCR quantification and revealed 66% *KRT10_arg_* (p9_arg_s_GFP) and 33% *KRT10_ala_* (p11_ala_s_GFP) products (Figure 1B).

In summary, the majority of identified mRNAs resulting from p4_var_g_GFP encoded an aberrant K10_arg_ (p6_arg_s_GFP, p8_arg_s_GFP, p9_arg_s_GFP). The remainder encoded either K10_ter_ (p14_ter_s_GFP) or K10_ala_ (p11_ala_s_GFP). This indicates that a change in the donor splice site of intron 6, due to the 1 bp deletion, results in alternative splicing and frequently arginine‐rich C‐termini. Alternative splicing is supported by splice site prediction tools such as BDGP (0.93 → below cut‐off 0.40; http://www.fruitfly.org/seq_tools/splice.html), NetGene2 (0.93 → 0.71; http://www.cbs.dtu.dk/services/NetGene2/) and SplicePort (1.06 → not recognized; http://spliceport.cbcb.umd.edu/). These suggested a reduced confidence for the recognition of the mutated splice site compared with the wild‐type sequence.

### 3.2 Nuclear translocation of K10 is initiated by an arginine‐rich C‐terminus

**Figure 2:**
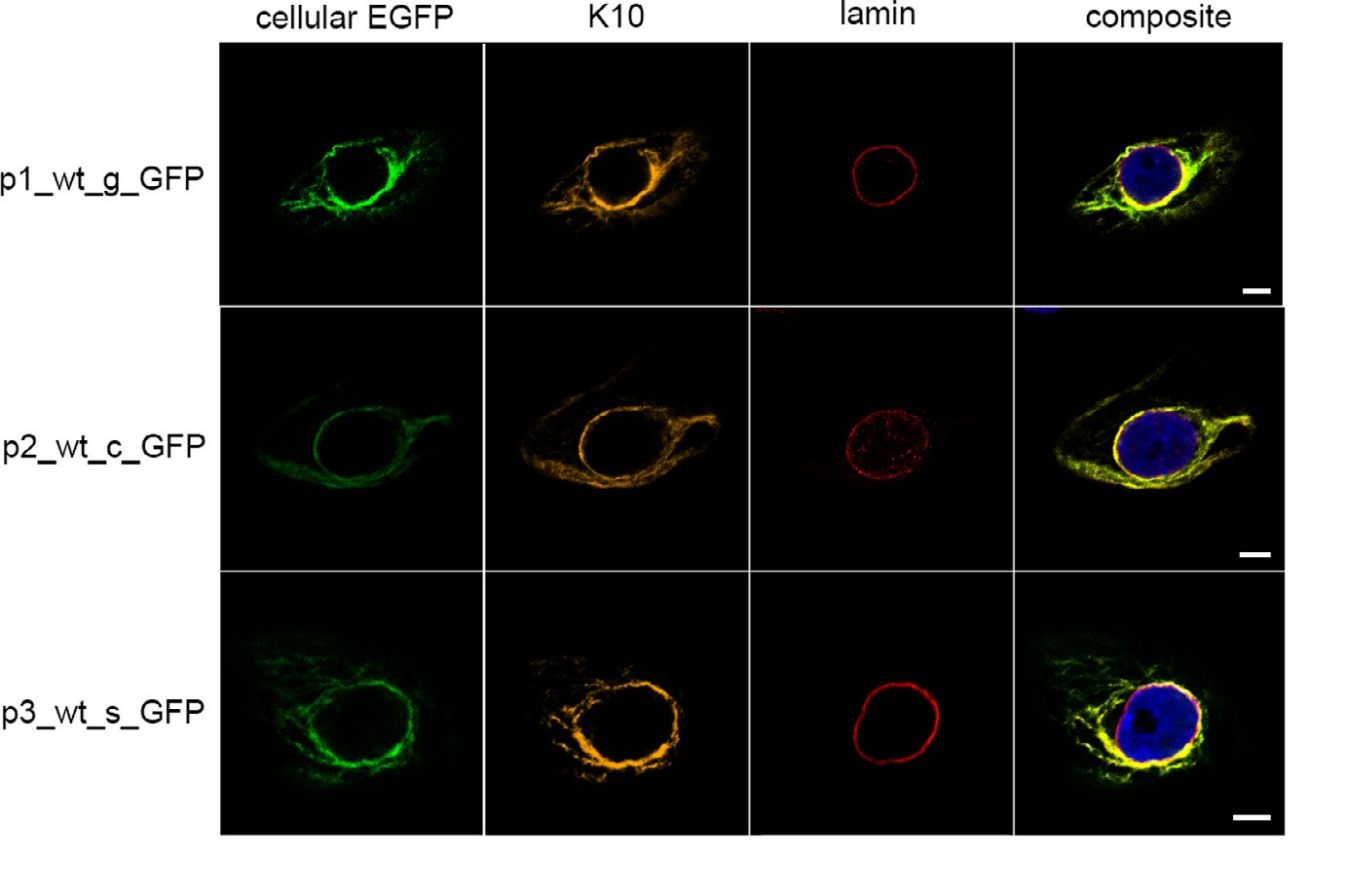
Localization of K10 following transient transfection of NKc21 keratinocytes with K10_wt_ encoding plasmids. K10 was detected either by imaging the eGFP fluorescence expressed from plasmid (cellular eGFP, green) or immunostaining with anti‐K10 antibody DE‐K10 (orange). The nuclear membrane was labelled with an anti‐lamin antibody (red). DAPI (blue) was used for nuclear staining. All K10 constructs: *KRT10* wild‐type gDNA (p1_wt_g_GFP), the corresponding cDNA (p3_wt_s_GFP) or cDNA from a wild‐type control (p2_wt_c_GFP) carried an N‐terminal eGFP‐tag. Antibodies against K10 and lamin showed clear cytoplasmic localization in every wild‐type K10. Scale bar, 5 μm

**Figure 3:**
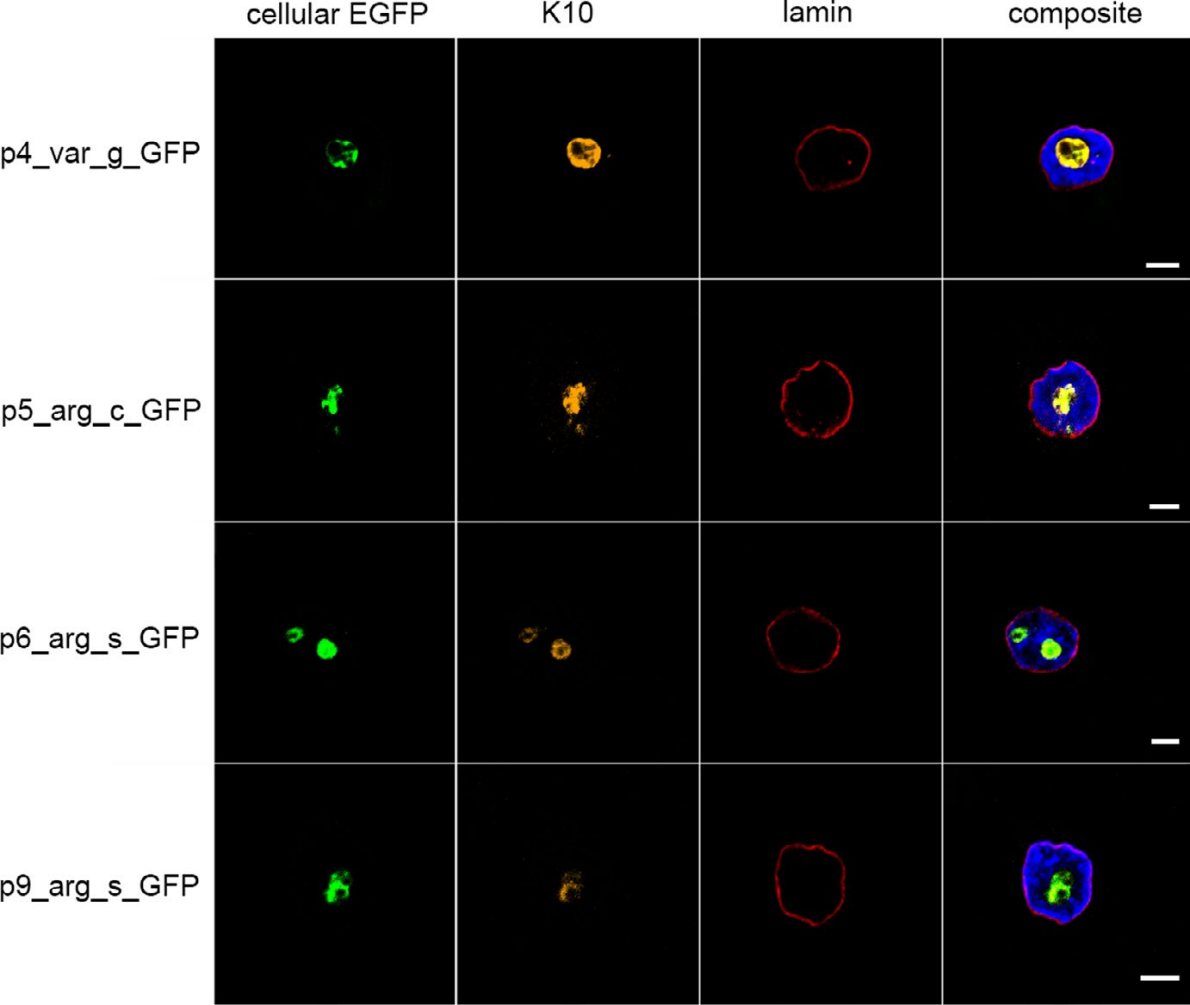
Localization of K10 following transient transfection of NKc21 keratinocytes with K10_arg_ encoding plasmids. K10 was detected either by imaging of eGFP expressed from plasmid (cellular eGFP, green) or immunostaining with an anti‐K10 antibody (DE‐K10, orange). The nuclear membrane was labelled with an anti‐lamin antibody (red). DAPI (blue) was used for nuclear staining. All K10 constructs: IWC‐causing *KRT10* gDNA (p4_var_g_GFP), the corresponding cDNA from splice products (p6_arg_s_GFP, p9_arg_s_GFP) and patient cDNA (p5_arg_c_GFP) carried an N‐terminal eGFP‐tag. The expressed K10 was localized in the nucleus of transfected keratinocytes. Scale bar, 5 μm

**Figure 4:**
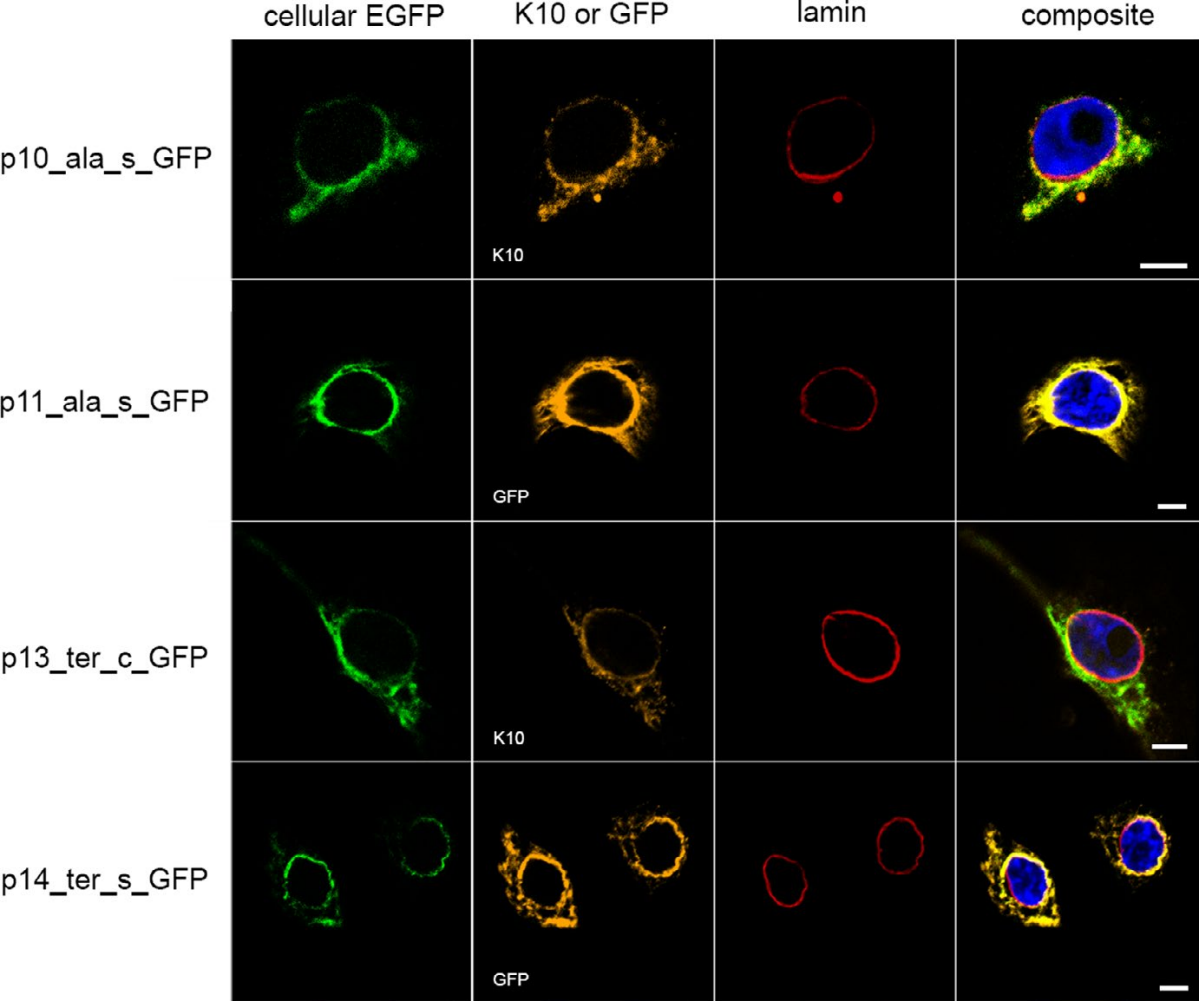
Localization of K10 following transient transfection of NKc21 keratinocytes with K10_ala_‐ or K10_ter_‐encoding plasmids. K10 was detected either by imaging of eGFP expressed from plasmid (cellular EGFP, green) or immunostaining with an anti‐K10 (DE‐K10) or anti‐GFP antibody (both orange). The nuclear membrane was labelled with an anti‐lamin antibody (red). DAPI (blue) was used for nuclear staining. All K10_ala_ (p10_ala_c_GFP, p11_ala_s_GFP) or K10_ter_ (p13_ter_c_GFP, p14_ter_s_GFP) carried an N‐terminal EGFP‐tag. K10_arg_ and K10_ter_ were both exclusively localized in the cytoplasm. As the aberrant K10 in p11_ala_s_GFP and p14_ter_s_GFP could not be detected with the DE‐K10 antibody, the anti‐GFP antibody was used for staining. Scale bar, 5 μm

The consequence of IWC‐associated *KRT10* variants on cellular localization was initially analysed in vitro. Plasmids encoding eGFP‐labelled *KRT10* cDNA were generated. These carried previously reported IWC‐associated frameshift variants described to result in each alternate reading frame (−2/+1 or −1/+2).^3^, ^11^, ^14^ Transient expression in keratinocytes resulted in arginine‐ (K10_arg_) and alanine‐rich (K10_ala_) K10 products, respectively. Additionally, keratinocytes transiently expressing a cDNA featuring a hypothetical nonsense variant localized to the region encoding the V2 domain of K10^3^, ^11^ were analysed. This resulted in the expression of a C‐terminal‐truncated K10 (K10_ter_). Alternatively, the plasmids carried a splice product expressed by NKc21 cells after transfection with p4_var_g_GFP (Table S1). Plasmids expressing these eGFP‐tagged *KRT10* cDNA variants (p2_wt_c_GFP, p5_arg_c_GFP, p10_ala_c_GFP, p13_ter_c_GFP) in addition to gDNA (p1_wt_g_GFP, p4_var_g_GFP) and previously subcloned splice products (p3_wt_s_GFP, p6_arg_s_GFP, p8_arg_s_GFP, p9_arg_s_GFP, p11_ala_s_GFP, p14_ter_s_GFP) were transiently transfected into NKc21 cells. This enabled analysis of the subcellular localization of each aberrant K10 product. Wild‐type K10 (K10_wt_) encoded by p1_wt_g_GFP, p2_wt_c_GFP and p3_wt_s_GFP was exclusively observed in the cytoplasm. Here, it was organized into filament structures (Figure 2). K10_arg_ from patient‐derived cDNA (p5_arg_c_GFP), K10_arg_‐expressing plasmids (p6_arg_s_GFP, p9_arg_s_GFP) and products translated from IWC‐related sequence (p4_var_g_GFP) displayed nuclear localization (Figure 3). K10_ala_ (p10_ala_c_GFP, p11_ala_s_GFP) and truncated K10_ter_ (p13_ter_c_GFP, p14_ter_s_GFP) were exclusively localized to the cytoplasm (Figure 4). As expected, aberrant K10 could not be detected via C‐terminus‐specific antibodies (EP1607IHCY, SP99) (Figure S2).

Co‐transfection with combinations of distinct fluorescence‐labelled *KRT10*_arg_ and *KRT10*_ala_ (p6_arg_s_GFP/p12_ala_s_mCherry or p7_arg_s_mCherry/p11_ala_s_GFP) resulted in an expected nuclear signal of K10_arg_. However, the unexpected nuclear localization of K10_ala_ was also observed (Figure S3). Co‐transfection with distinct fluorescence‐labelled K10_arg_ (p7_arg_s_mCherry) and K10_wt_ (p3_wt_s_GFP) resulted in nuclear‐localized eGFP. This indicated co‐translocation of K10_wt_ with K10_arg_ into the nucleus. Further, it may suggest the nuclear translocation of higher keratin polymers (Figure S3).

Immunostaining of cellular K5 and K14 after NKc21 transfection with a K10_arg_ vector (p5_arg_c_GFP) revealed a nuclear signal for endogenous K5 in approximately 30% of all transfected cells (Figure S4). K5 is a type II keratin, a promiscuous assembly partner of the type I K10. The apparent nuclear signal of K5 suggests the co‐transport of keratin dimers. A nuclear K14 signal was not detectable (data not shown).

Our results indicate that exclusively aberrant K10_arg_ is translocated to the nucleus. Furthermore, they suggest that aberrant K10_arg_ can pull other keratins (ie K10_wt_, K10_ala_ and K5), which do not feature an arginine‐rich C‐terminus, into the nucleus. This likely occurs as keratin dimers and tetramers.

### 3.3 Endogenous expression of aberrant K10 leads to abnormal differentiation of keratinocytes in culture

Keratinocyte clones were generated via CRISPR/Cas9‐mediated gene editing enabling the introduction of heterozygous −2/+1 (*KRT10_arg_*) or −1/+2 (*KRT10_ala_*) frameshifts into regions encoding the V2 domain. As a result, variants similar to those found in IWC patients were generated in NKc21 keratinocytes. This enabled the analysis of aberrant K10 subcellular localization in an endogenous setting. Six clones with heterozygous frameshift variants were studied. Four displayed expression K10_arg_ (e1_arg_33‐1, e2_arg_33‐1, e3_arg_26‐1, e4_arg_26‐1), and two demonstrated expression K10_ala_ (e5_ala_26‐1, e6_ala_26‐1) (Table S2). Sequencing of cDNA derived from differentiated cells confirmed transcription of the expected *KRT10* mRNAs (Table S2).

**Figure 5:**
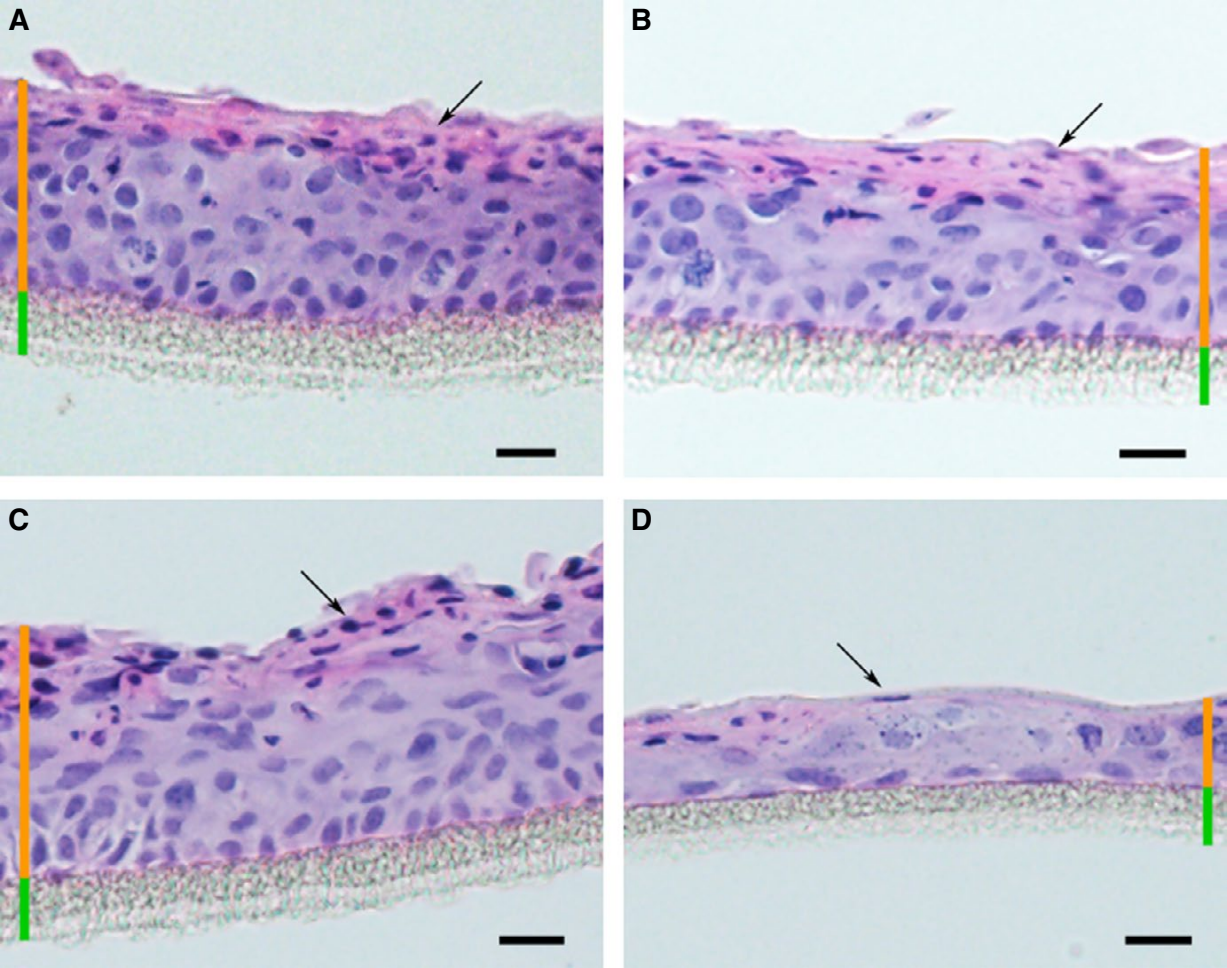
Epidermal equivalents derived from gene edited NKc21 single‐cell clones expressing K10_ala_ displayed thin epithelium. Haematoxylin and eosin staining of epidermal models. Epidermal equivalents derived from wild‐type (A), mock control (B), and heterozygous K10_arg_ clones (C) displayed a differentiated epithelium including basal layer and stratum spinosum (orange bar). Epidermal equivalents derived from K10_ala_ clones demonstrated a thin epithelium (D). All shown clones were cultivated in parallel, epidermal equivalents C and D are derived from clones e4_arg_26‐1 (K10_arg_) and e6_ala_26‐1 (K10_ala_). Parakeratosis was observed in all samples (black arrows). The supporting filter membrane is indicated (green bar). Scale bar (black), 20 μm

Epidermal equivalents derived from K10_arg_ single‐cell clones were subjected to haematoxylin and eosin (HE) staining. These displayed stratified epithelium including: basal layer, differentiated suprabasal layers and stratum corneum comparable to wild‐type and mock controls (Figure 5A‐C). However, epidermal equivalents derived from K10_ala_ clones displayed an epithelium with strongly impaired differentiation and significantly thinner than epidermal equivalents from K10_arg_ and K10_wt_ (Figure 5D, Figure S5).

**Figure 6:**
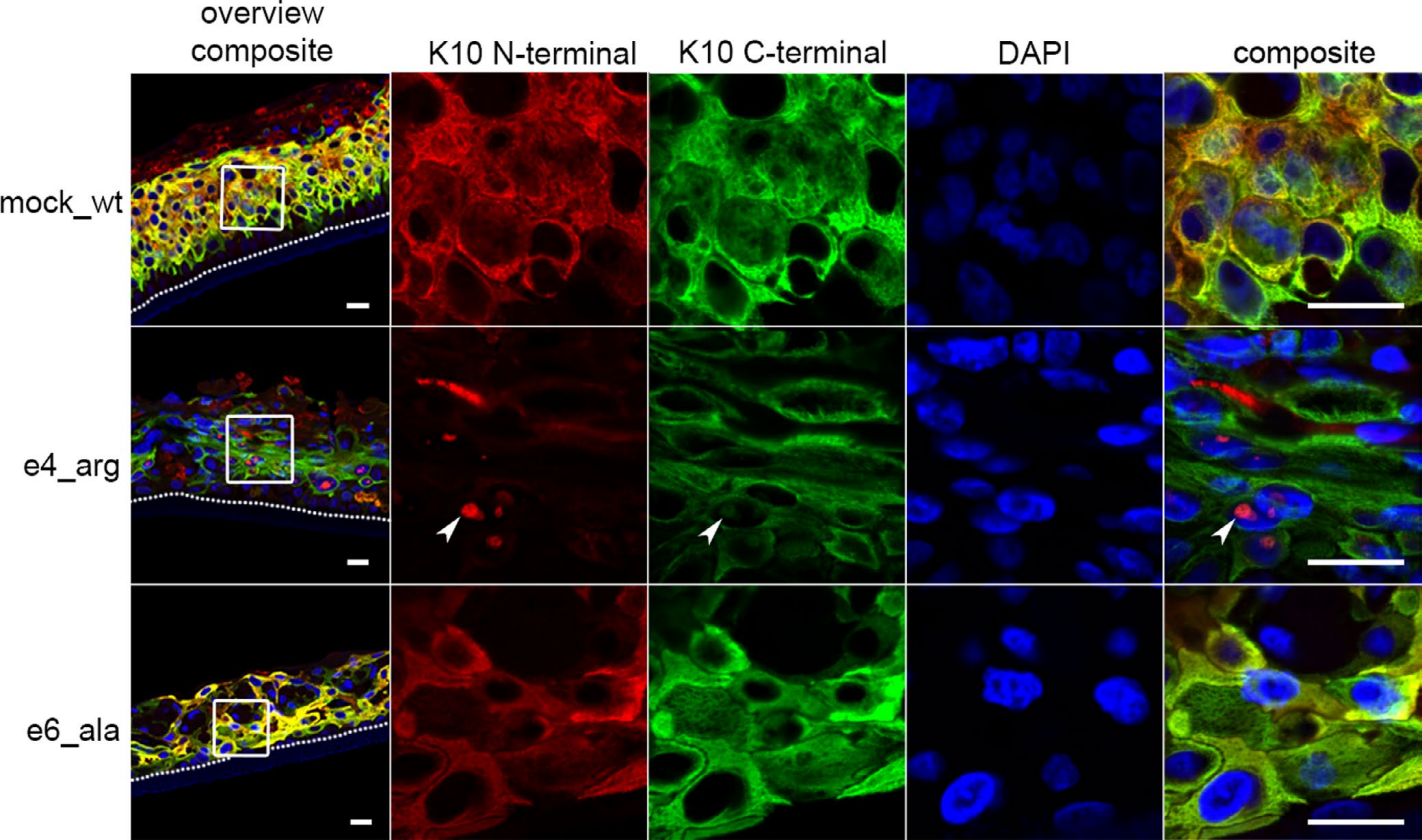
Epidermal equivalents derived from K10_arg_ keratinocyte single‐cell clones display nuclear localization of K10 in differentiated suprabasal layers. Immunofluorescence staining via N‐ (LH2, exact binding region unknown; red) and C‐terminus‐specific (EP1607IHCY, amino acids 555‐584; green) K10 antibodies indicated nuclear localization of K10 in the suprabasal keratinocytes of K10_arg_‐derived equivalents (white arrowhead). In K10_wt_‐ or K10_ala_‐derived equivalents, K10 was localized exclusively in the cytoplasm. Nuclear co‐translocation of endogenous K10_wt_ and K10_arg_ (e4_arg_26‐1) enabled observation of nuclear localization via the C‐terminus‐specific anti‐K10 (white arrowhead). DAPI (blue) was used for nuclear staining. Border between supporting filter membrane and basal layer indicated (white‐dotted line). Scale bar, 20 μm

Immunofluorescence analysis of differentiated keratinocytes via either N‐ or C‐terminus‐specific anti‐K10 antibodies indicated a nuclear localization of K10 signal in K10_arg_ keratinocytes. K10_ala_‐differentiated keratinocytes displayed cytoplasmic signals alone, comparable to K10_wt_ (Figure 6). Immunostaining via the C‐terminus‐specific K10 antibody enabled exclusive staining of K10_wt_. This revealed nuclear localization of K10_wt_ in the differentiated suprabasal layers of epidermal equivalents derived from heterozygous K10_arg_ keratinocyte single‐cell clones. Similar observations were noted following co‐immunostaining for K5. This supports our conclusion that, minimally, keratin tetramers driven by K10_arg_ are subjected to nuclear co‐localization (Figure S6). Additionally, lamin B1 displayed reduced expression in K10_ala_ expressing keratinocytes. This may indicate a disturbed nuclear integrity in affected keratinocytes.

## 4 DISCUSSION

In keratinocytes derived from IWC patients, K10 displays nuclear localization, correlated with −2/+1 frameshift variants (K10_arg_). However, the effect of K10_ala_ was previously inconclusive. Approximately 70% of IWC patients carry *KRT10* frameshift variants. These affect splice sites in 60% of cases. Of the currently reported IWC‐associated *KRT10* splice site variants, approximately 30% affect the donor splice site of intron 6. Analysis of mRNA from patients with IWC‐associated splice site variants revealed transcripts that would be translated into K10 with arginine‐rich tails.^1^ Lim et al^10^ described a K10 splice site variant, comprised of a 1 bp deletion at the exon‐intron boundary of exon 6/IVS6. They suggested that this variant resulted in K10_ala_ expression, without experimental confirmation.^10^ In the present study, we demonstrated that this variant leads to several alternative splicing products. In contrast to prior suggestions, the majority of these products are predicted to result in K10_arg_ expression.

C‐terminal frameshift variants in *KRT10* remain poorly investigated in a systematic manner. Therefore, little is understood regarding the effects of IWC‐associated variants on the cellular localization of K10. Our studies provide the first systematic analysis on nuclear K10 localization in IWC. Three possible C‐terminal frames of *KRT10* were analysed in addition to a speculative nonsense variant. Our data clearly indicate that only K10_arg_ translocates to the nucleus, whereas K10_wt_, K10_ala_ and K10_ter_ remain in the cytoplasm. However, the presence of K10_arg_ enables the co‐translocation of non‐K10_arg_ into the nucleus.

These results contradict a previous suggestion that K10_ala_ may cause pathogenicity in an IWC patient with a splice site variant.^10^ However, sequencing of the corresponding cDNA was not previously performed to confirm a frameshift at the level of transcription. Human splice sites are highly conserved; >99.9% are canonical (donor site: GT, GC, AT).^23^ Further, exonic sequences close to the exon‐intron boundaries are highly conserved. The 3′‐most exonic nucleotide is a guanine in over 80% of instances.^24^, ^25^, ^26^ Analysis of splicing products from gDNA harbouring this variant revealed five transcripts. Three of these encoded K10_arg_, one encoded K10_ter_, and a minor transcript encoded K10_ala_ at the limit of detection. The presence of these transcript varieties concurs with splice site prediction tools. However, a similar in vivo splicing profile could only be confirmed in patient epidermal biopsies.

In addition to the patient described by Lim et al,^10^ three families reportedly carry similar variants with exchanges in the splice site sequence (c.1373+1G>A; c.1373+1G>C; c.1373+2T>C).^1^, ^11^ mRNA isolated from a patient carrying the c.1373+1G>A variant displayed a full deletion of exon 6,^1^ a product identical to a major splice product that we observed (p6_arg_s). This patient data support our findings that splicing of this mutant mRNA frequently causes deletion of exon 6. This results in frameshift and consequently K10_arg_ expression.

In K10_ala_, altered C‐terminal amino acids result in the loss of neutral glycine loops in favour of neutral alanine sequences. Basic amino acids, associated with classical nuclear localization sequences, are not present.^27^ As a result, K10_ala_ is not expected to display an altered subcellular localization. In contrast, the basic amino acid arginine is assumed to change the pK‐value of the C‐terminus of K10_arg_ and subsequently the recognition signal for nuclear targeting.^28^

The endogenous model, described in the present study, supported a cytoplasmic localization of K10_ala_. Epidermal equivalents grown from keratinocytes expressing K10_ala_ demonstrated significantly impaired stratification. In contrast to K1, in which an alanine‐rich C‐terminus results in the severe phenotype of IHCM^15^, ^16^, ^29^ or SPPK,^17^ no patients have been described with K10_ala_‐associated variants. This is in line with our data of an impaired differentiation in K10_ala_ keratinocytes, which might result in lethality.

Keratins promiscuously form heterodimers consisting of equimolar amounts of type I and type II keratins.^6^ These subsequently polymerize to form keratin IFs. K1 is present within the nucleus of K10_arg_‐positive keratinocytes of IWC patients.^1^ This is likely co‐translocated with K10_arg_ in the form of dimers. In line with this observation, we observed nuclear co‐localization of K5 with K10_arg_ in our model systems. This has also been observed in patient keratinocytes (unpublished data). Further, we observed nuclear co‐localization of K10_arg_/K10_wt_ in addition to K10_arg_/K10_ala_. This may be a result of K10_arg_‐containing tetramers or higher‐order polymers passing the nuclear membrane. However, K14 was unexpectedly not detectable in the nuclear keratin complexes, possibly a result of poor epitope recognition by the monoclonal antibody.

In summary, this study provides a deeper understanding of the nuclear localization of aberrant K10 in IWC which is correlated to a single altered reading frame in K10. We confirmed that arginine‐rich K10 tails, and not alanine‐rich K10 tails, are responsible for the pathogenic nuclear localization of K10 in affected patients. A greater understanding of keratin nuclear localization in IWC advances the potential use of the subsequent mechanism of chromosomal exchange for natural gene therapies.

## CONFLICT OF INTEREST

Nothing to disclose.

## AUTHOR CONTRIBUTIONS

PR, EI, IS, HW and SvA performed the experiments. MA, OPM and AV contributed essential tools. PR, AV and BB analysed the data. BB, JR and OPM wrote the paper. PHI, JR and BB designed the study.

## Data Availability

I am willing to share my research data.
