# Supplementary figures and images for: Arginine‐ but not alanine‐rich carboxy‐termini trigger nuclear translocation of mutant keratin 10 in ichthyosis with confetti
Source: J Cell Mol Med. 2019 Oct 22;23(12):8442–52. doi: 10.1111/jcmm.14727 (PMC6850952; doi:10.1111/jcmm.14727)

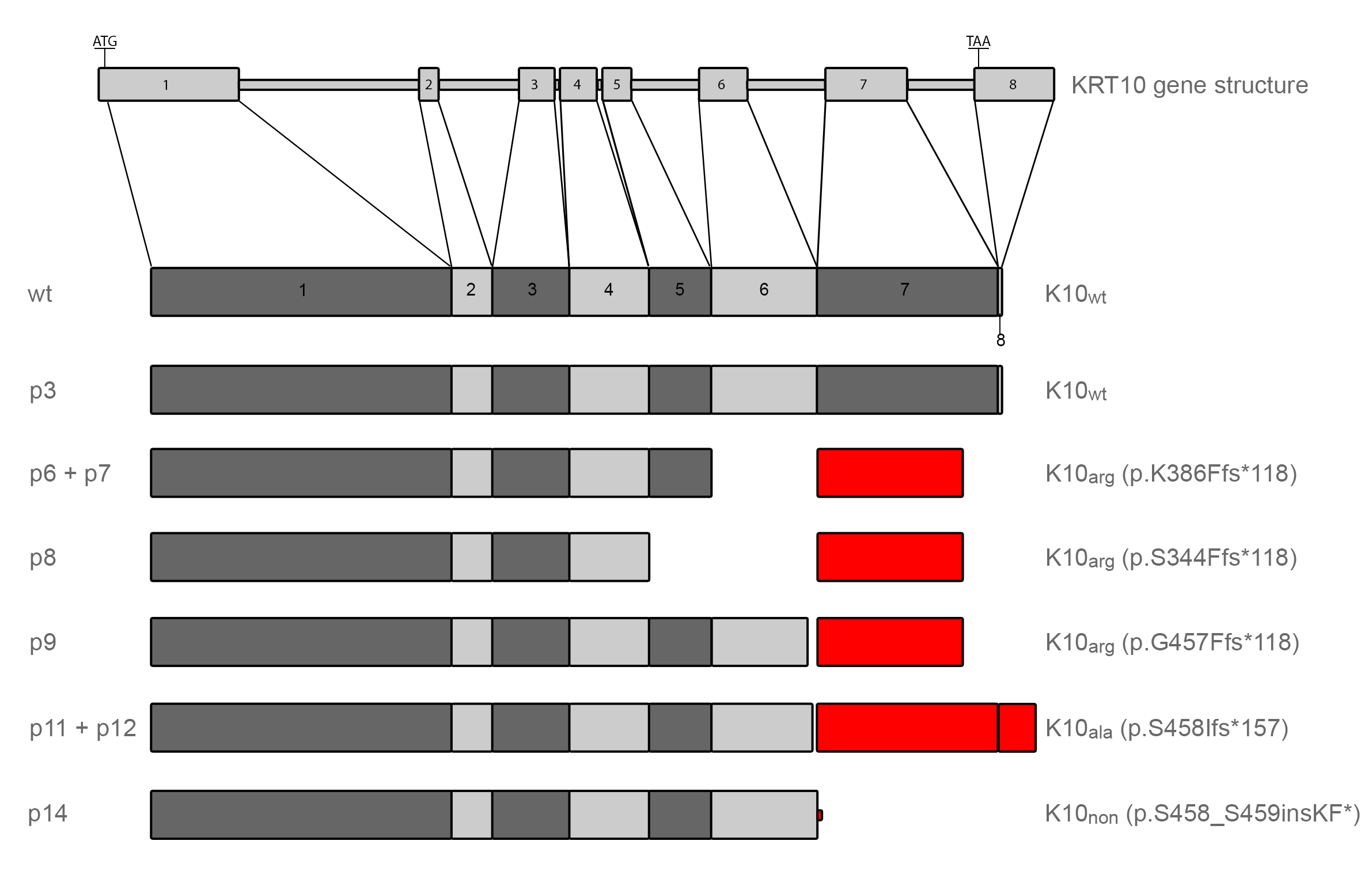

Supplement: Supplementary file 1 [file JCMM-23-8442-s001.tif]

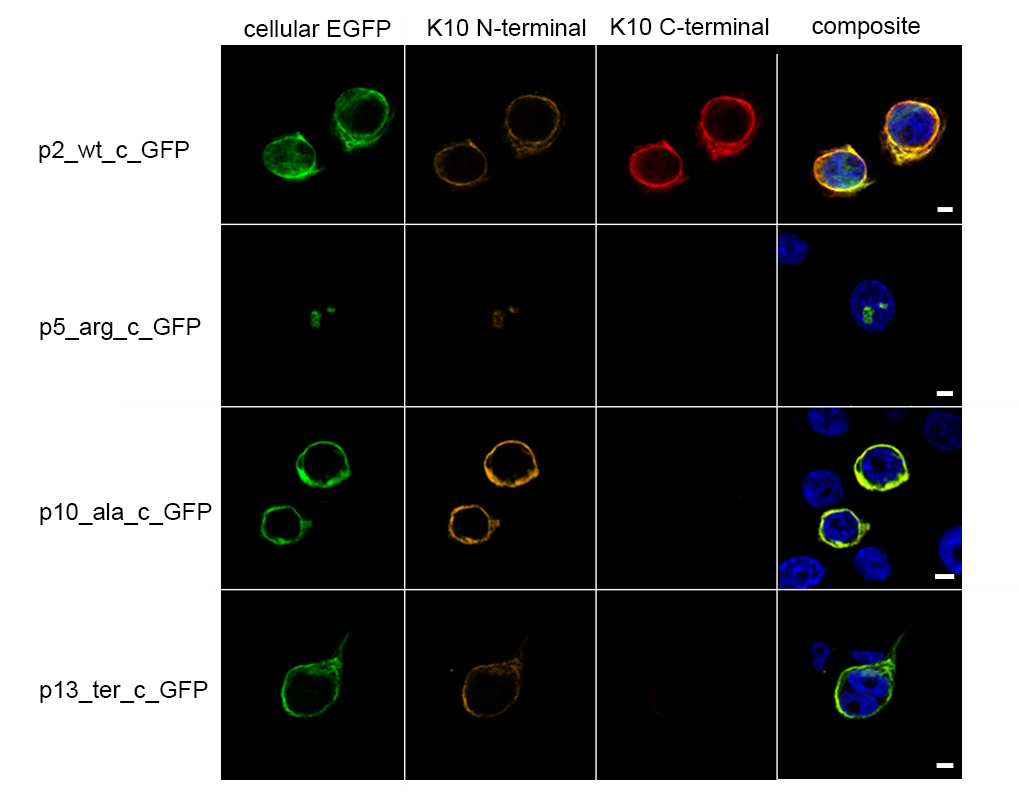

Supplement: Supplementary file 2 [file JCMM-23-8442-s002.tif]

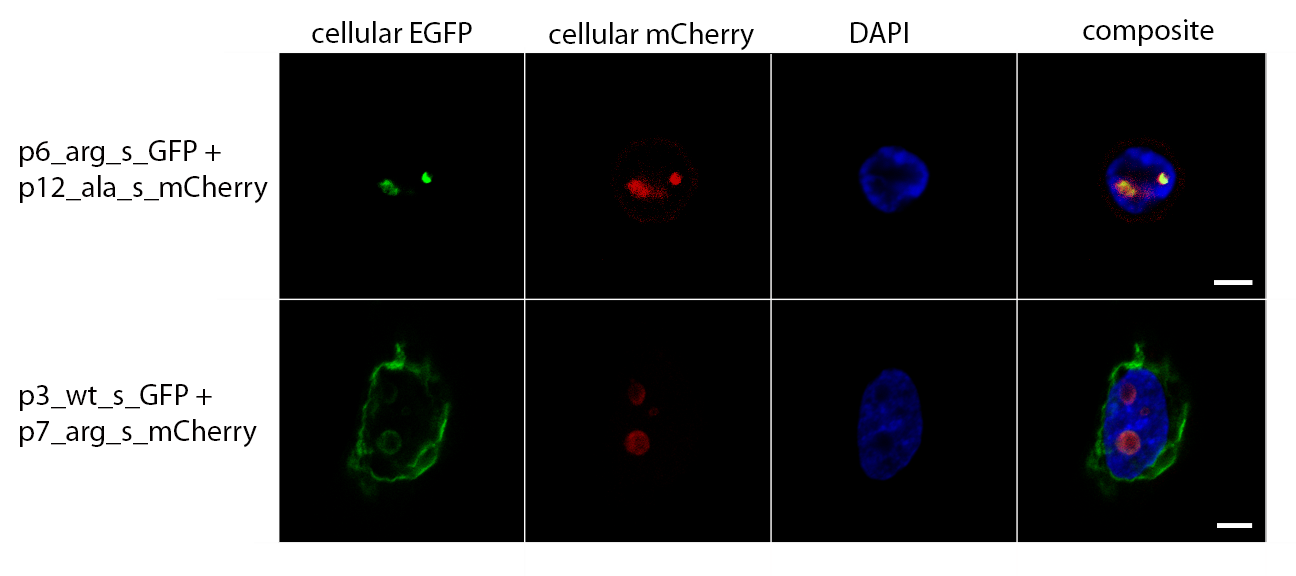

Supplement: Supplementary file 3 [file JCMM-23-8442-s003.tif]

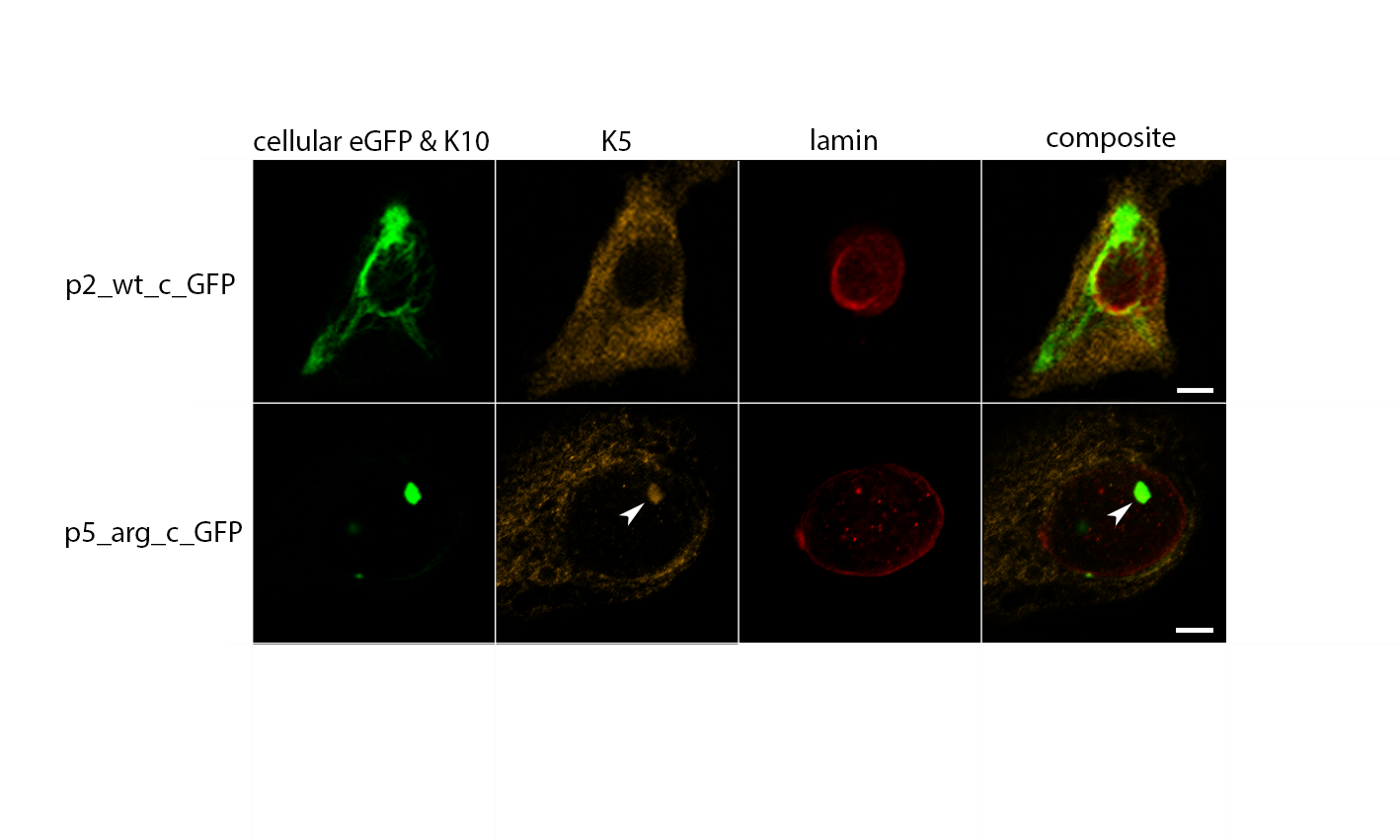

Supplement: Supplementary file 4 [file JCMM-23-8442-s004.tif]

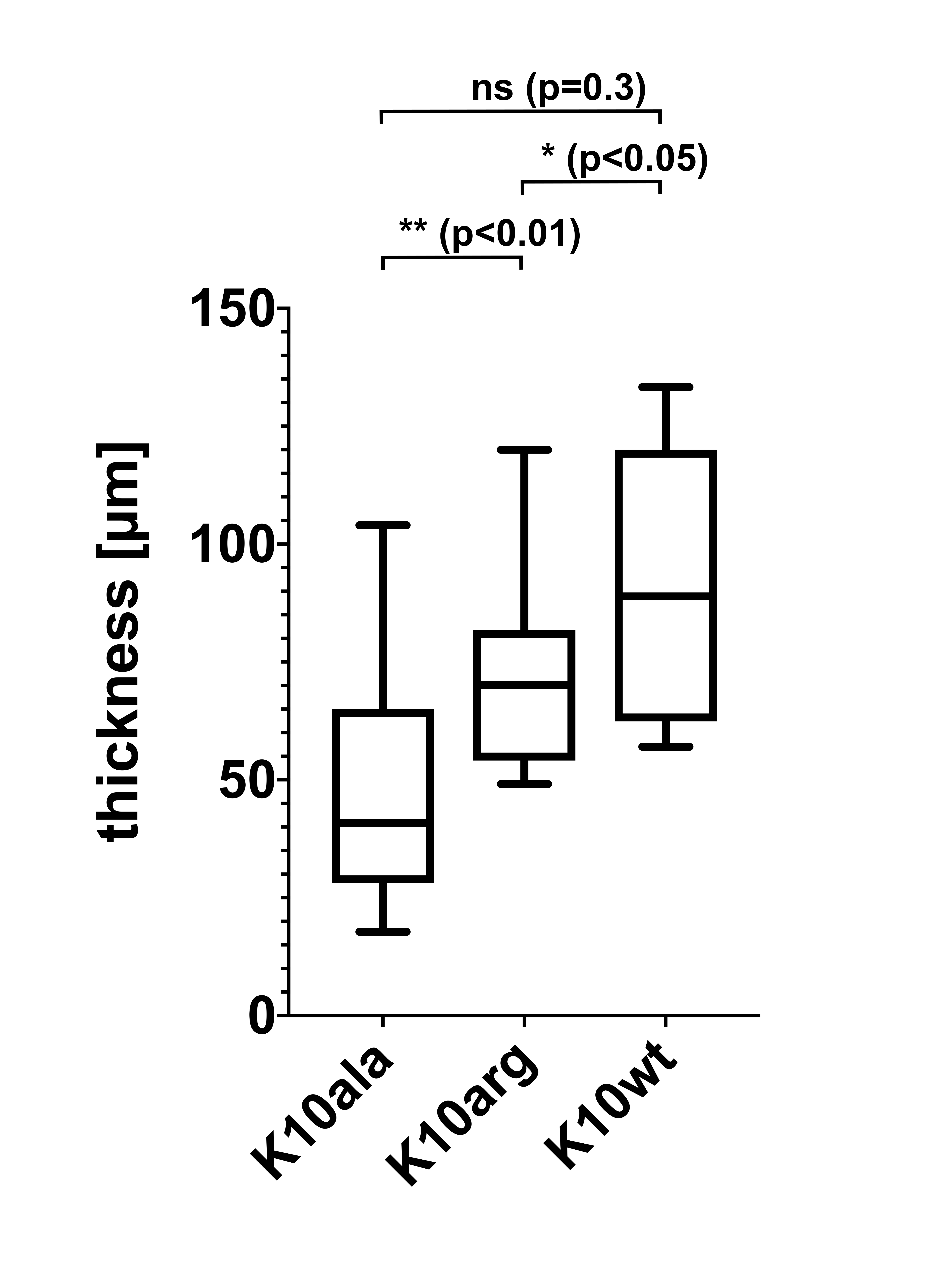

Supplement: Supplementary file 5 [file JCMM-23-8442-s005.tif]

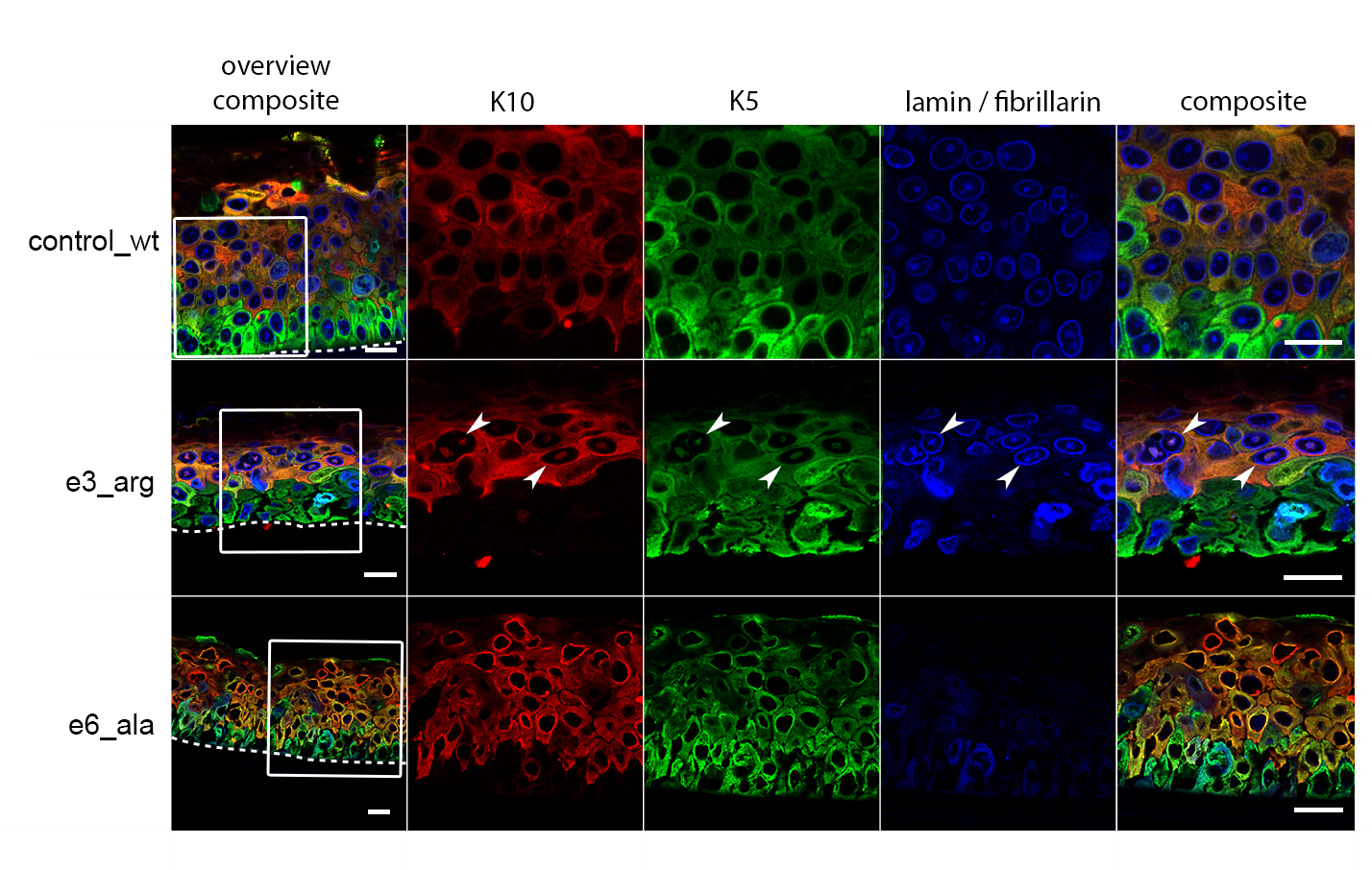

Supplement: Supplementary file 6 [file JCMM-23-8442-s006.tif]
