# Supplementary material for: Arginine‐ but not alanine‐rich carboxy‐termini trigger nuclear translocation of mutant keratin 10 in ichthyosis with confetti
Source: J Cell Mol Med. 2019 Oct 22;23(12):8442–52. doi: 10.1111/jcmm.14727 (PMC6850952; doi:10.1111/jcmm.14727)
Supplement: Supplementary file 7 [file JCMM-23-8442-s007.docx]

Table S1. Expression plasmids were generated, featuring various modified cDNA or gDNA *KRT10* sequences. All *KRT10* sequences, except those from p2, p5, p10, p13, additionally showed a common polymorphism of a 12 bp deletion (p.Gly490_Gly493del) (rs778613907) (MAF 0.2617) (Burger et al. 2012). This enables distinction between plasmid/plasmid-derived and endogenous genomic sequences. Plasmid names: type of K10 C-terminus (wt = wildtype, arg = arginine-rich, ala = alanine-rich, ter = premature stop, var = inserted variation)_source of inserted sequence (g = genomic DNA, c = cDNA, s = cDNA after *in vitro* splicing)_type of N-terminal tag (GFP = eGFP, mCherry).

| **plasmid name** | **inserted DNA type** | **C-terminus K10** | ***KRT10* sequence cDNA / RNA (NM_000421)** | **consequence of change** | **changes at protein level (NP_000412.3)** | **original name** | **reference** |
| --- | --- | --- | --- | --- | --- | --- | --- |
| p1_wt_g_GFP | gDNA | wildtype | wildtype | none | none | p103 |  |
| p2_wt_c_GFP | cDNA | wildtype | wildtype | none | none | p81 |  |
| p3_wt_s_GFP | cDNA | wildtype | wildtype | none | none | p122 |  |
| p4_var_g_GFP | gDNA | splice site variant^‡^ | c.1373+1delG^‡^ | see p6-p8, p11, p14 | see p6-p8, p11, p14 | p108 | Lim et al., 2016 |
| p5_arg_c_GFP | cDNA | arginine-rich | c.1546_1551delinsT | deletion of 5 bp in exon 7 | p.Gly516PhefsTer63 | p83 | Burger et al., 2012 |
| p6_arg_s_GFP | cDNA | arginine-rich | r.1156_1373del218 | deletion exon 6 | p.Lys386PhefsTer118 | p113 |  |
| p7_arg_s_mCherry | cDNA | arginine-rich | r.1156_1373del218 (=p6) | deletion exon 6 (=p6) | p.Lys386PhefsTer118 | p128 |  |
| p8_arg_s_GFP | cDNA | arginine-rich | r.1030_1373del44 | deletion exon 5 and 6 | p.Ser344PhefsTer118 | p114 |  |
| p9_arg_s_GFP | cDNA | arginine-rich | r.1369_1373del5 | deletion of last 5 bp of exon 6 | p.Gly457PhefsTer118 | p116 |  |
| p10_ala_c_GFP | cDNA | alanine-rich | c.1544delG | deletion of 1 bp in exon 7 | p.Gly514AlafsTer104 | p85 | Hotz et al., 2016a^††^ |
| p11_ala_s_GFP | cDNA | alanine-rich | r.1373delg | deletion of last 1 bp of exon 6 | p.Ser458IlefsTer157 | p117 |  |
| p12_ala_s_mCherry | cDNA | alanine-rich | r.1373delg (=p11) | deletion of last 1 bp of exon 6 | p.Ser458IlefsTer157 | p129 |  |
| p13_ter_c_GFP | cDNA | nonsense | c.1506_1507insCT | stop codon in exon 7 | p.Ser503Ter | p86 |  |
| p14_ter_s_GFP | cDNA | inframe insertion | r.[1373del;1373_1374ins1373+1_1373+355] | insertion IVS 6 | p.Ser458_Ser459insLysPheTer | p111 |  |

^‡^ originally described as c.1373delG (alanine-rich carboxy-terminus)

^††^ initial description of mutation

Table S2. Two distinct sgRNAs targeting exon 6 (33) and exon 7 (26) of *KRT10* were generated for gene editing. Identical editing experiments are indicated with the same number following the designated sgRNA identity. Six clones carried a heterozygous deletion within *KRT10* (e1-e6). Four clones carried K10_arg_-associated mutations (e1-e4) and two clones carried K10_ala_-associated mutations (e5, e6). The majority of clones displayed additional inframe indel mutations, many on the alternate allele.

| **clone name** | **C-terminus K10** | **genotype of *KRT10* alleles** | **frameshift causing mutation** | **K10 protein** | **additional inframe alterations** | **original name** |
| --- | --- | --- | --- | --- | --- | --- |
| e1_arg_33-1 | arginine-rich | + / mut | c.1367_1368delAG | p.Glu456GlyfsTer122 | c.1362_1367delAGGGAG; p.Glu454_Glu456delinsGlu | Η1 |
| e2_arg_33-1 | arginine-rich | + / mut | c.1367_1368delAG | p.Glu456GlyfsTer122 | c.1362_1367delAGGGAG; p.Glu454_Glu456delinsGlu | Η3 |
| e3_arg_26-1 | arginine-rich | + / mut | c.1408_1514del107bp | p.Gly470ArgfsTer85 | c.1409_1411GCG>TAA  p.Gly470_Gly471delinsValSer | Δ10 |
| e4_arg_26-1 | arginine-rich | + / mut | c.1409dupG | p.Gly470GlyfsTer121 | c.1395_1592del198;  p.Gly466_Ser531del | Δ25 |
| e5_ala_26-1 | alanine-rich | + / mut | c.1409_1514del106bp | p.Gly470AlafsTer124 | none | Δ3 |
| e6_ala_26-1 | alanine-rich | + / mut | c.1409delG | p.Gly470AlafsTer159 | c.[1398_1400delCGG]; [1398_1400delCGG,1414_1416delGGC]  p.[Gly467del];[Gly467del, p.Gly472del] | Δ12 |

Table S3. Primer sequences used in the present study for plasmid generation, sequencing of *KRT10* mRNAs, and quantification of ratio between p9_arg_s_GFP and p11_ala_sGFP.

| **primers** | **sequences** | **assays** |
| --- | --- | --- |
| 751-XhoI-KRT10-F | ctgactcgagcgATGtctgttcgatacagctc | generation of p2, p5, pUC-K10 (intermediate plasmid generation of p1) |
| 752-KRT10-HindIII-R | taagtaaagcttttaatagtagtgtttcttgg | generation of p2, p5 |
| 290-KRT10-R | gcattgcatattcttaggtgagc | generation of pUC-K10 (intermediate plasmid generation of p1) |
| 819-KRT10-F | ttgatctgactcaacttctg | generation of pUC_K10-ex5-3’ (intermediate plasmid generation of p1) |
| 876-KRT10-HindIII-R | agtaaagctttccatagaccatcaagacag | generation of pUC_K10-ex5-3’ (intermediate plasmid generation of p1) |
| 733-eGFP-F | GGCATGGACGAGCTGTACAAG | subcloning cDNA p3, p6, p8, p9, p11, p14 |
| 900-MCS-F | CTCTACAAATGTGGTATGGCTG | subcloning cDNA p3, p6, p8, p9, p11, p14 |
| 737FAM-F | FAM-GGATATTAAGATCCGACTGGAG | quantitative assessment p9_arg_s_GFP vs p11_ala_s_GFP |
| 294b-KRT10-R | GGAACTGCCACCACCGTAG | quantitative assessment p9_arg_s_GFP vs p11_ala_s_GFP |
| 963-AgeI-mCherry-F | gtcaccggtccATGGTGAGCAAG | exchange eGFP and mCherry |
| 964-mCherry-XhoI-R | cgtcctcgagaCTTGTACAGCTCGTCCATGCC | exchange eGFP and mCherry |

Table S4. Primary and secondary antibodies.

| **primary antibody** | **name** | **antigen** | **company** |  | **dilution** |
| --- | --- | --- | --- | --- | --- |
| Rabbit serum |  | **K1**, aa 576-625 | Abcam | #ab83664 | 1:1000 |
| Guinea pig serum |  | **K1**, aa 21-33 | Progen | #GP-K1 | 1:100 |
| Guinea pig serum |  | **K5**, C-terminus | Progen | #905501 | 1:100 |
| Mouse monoclonal | DE-K10 | **K10**, N-terminus | Progen | #11414 | 1:100 |
| Rabbit monoclonal | SP99 | **K10**, aa 468-584 | Abcam | #ab183317 | 1:200 |
| Rabbit monoclonal | EP1607IHCY | **K10**, aa 555-584 | Abcam | #ab76318 | 1:250 |
| Rabbit monoclonal | EP1612Y | **K14**, aa 400-500 | Abcam | #ab51054 | 1:100 |
| Goat serum |  | **GFP** | Abcam | #ab5450 | 1:1000 |
| Rabbit serum |  | **fibrillarin**, aa 1-100 | Abcam | #5821 | 1:1000 |
| Rabbit serum |  | **lamin B1**, aa 400-500 | Abcam | #ab16048 | 1:800 |

| s**econdary antibody** | **conjugated fluorophore** | **company** |  | **dilution** |
| --- | --- | --- | --- | --- |
| anti-guinea pig IgG | Alexa Fluor 594 | Jackson ImmunoResearch | #706-585-148 | 1:250 |
| anti-mouse IgG | Alexa Fluor 488 | Jackson ImmunoResearch | #715-545-150 | 1:500 |
| anti-mouse IgG | Alexa Fluor 594 | Life Techologies | #A21203 | 1:200 |
| anti-rabbit IgG | Alexa Fluor 488 | Jackson ImmunoResearch | # 711-545-152 | 1:500 |
| anti-rabbit IgG | Alexa Fluor 647 | Jackson ImmunoResearch | #711-175-152 | 1:500 |
| anti-mouse IgG | Alexa Fluor 647 | Jackson ImmunoResearch | #715-605-150 | 1:400 |

**Legend of supplemental figures**

**Figure S1. Plasmid constructs harbouring eGFP / mCherry-tagged *KRT10* cDNAs carrying C-terminal frameshift variants result in K10 variants.** Constructs were generated from mRNA transcribed in NKc21 keratinocytes following transient transfection with either wildtype (p1_wt_g_GFP) or IWC-associated (p4_var_g_GFP) *KRT10* gDNA. The p3_wt_s_GFP product, spliced from p1, was comparable to the natural wildtype product. All splice products from p4 transcribed altered K10 with either premature stop (p14_ter_s_GFP), an arginine-rich (p6_arg_s_GFP/p7_arg_s_mCherry, p8_arg_s_GFP, p9_arg_s_GFP) or an alanine-rich C-terminus (p11_ala_s_GFP/p12_ala_s_mCherry). Indicated in red are the changed K10 C-termini.

**Figure S2. N- and C-terminus-specific immunofluorescence staining of K10 in NKc21 keratinocytes transiently transfected with K10 variant-encoding plasmids.** NKc21 cells were transiently transfected with plasmids expressing cDNA of a wildtype control (K10_wt_; p2_wt_c_GFP), an IWC patient (K10_arg_; p5_arg_c_GFP), a previously described alanine-shift (K10_ala_; p10_ala_c_GFP), or a hypothetical K10_ter_ (p13_ter_c_GFP). K10 was detected either by imaging of eGFP expressed from plasmid (cellular EGFP, green) or immunostaining with either an N- (DE-K10, orange) or C-terminus-specific anti-K10 antibody (EP1607IHCY, red). K10_wt_ could be detected via both antibodies. All aberrant K10 could only be detected by the N-terminus-specific antibody, confirming the deleterious effect of the respective mutations on the K10 tail domain. Similar results were obtained using anti-K10 antibodies LH2 (N-terminal binding) and SP99 (C-terminal binding) (data not shown). DAPI (blue) was used for nuclear staining. Scale bar, 5 μm.

**Figure S3. Nuclear co-localization of arginine-rich K10 with alanine-rich and wildtype K10.** NKc21 cells were transiently co-transfected with either p6_arg_s_GFP and p12_ala_s-mCherry or p3_wt_S_GFP and p7_arg_s_mCherry. The nuclear localization of K10_arg_, K10_wt_ and K10_ala_ was detected by imaging of the eGFP (cellular eGFP, green) and mCherry (cellular mCherry, red). Overlapping fluorescence of K10_arg_ and either K10_ala_ or K10_wt_ confirmed nuclear transport of higher polymers. DAPI (blue) was used for nuclear staining. Scale bar, 5 μm.

**Figure S4. Nuclear co-localization of arginine-rich K10 with endogenous K5.** NKc21 cells were transiently transfected with either p1_wt_g_GFP or p5_arg_c_GFP. Co-staining of endogenous K5 (GP-CK5, orange) and K10 (cellular eGFP and DE-K10, both green) indicated a nuclear K5 co-localization with K10 in K10_arg_-expressing cells (arrow). Staining of lamin (red) confirmed this nuclear localization. Scale bar, 5 μm.

**Figure S5. Quantitation of epidermal thickness was calculated on hematoxylin and eosin stained epidermal equivalents.** At least four distinct areas were measured per epidermal equivalent. Six differentiated K10_arg_ (2x e3_arg_26-1, 4x e4_arg_26-1), seven differentiated K10_ala_ (4x e5_ala_26-1, 3x e6_ala_26-1), and two K10_wt_ clones were included. Ratio between thickness of keratinocyte layers and insert membrane was calculated to avoid bias by embedding technique e.g. cutting angle. Brown-Forsythe and Welch ANOVA tests resulted in significant differences in thickness between the K10_ala_ and K10_arg_ or K10_wt_ epidermal equivalents.

**Figure S6.** **Epidermal equivalents derived from K10_arg_ keratinocyte single cell clones display nuclear co-localization of K10 and K5 in differentiated suprabasal layers.** Co-staining of endogenous K5 (GP-CK5, green) and K10 (DE-K10, red) indicated a nuclear K5 co-localization with K10 in the differentiated suprabasal layers of K10_arg_-derived epidermal equivalents (arrow). In K10_wt_- and K10_ala_-derived keratinocytes, K10 and K5 were localized exclusively in the cytoplasm. Lamin B1 staining indicates the nuclear membrane and fibrillarin staining confirmed the nuclear localization (both blue). Scale bar, 20 μm.
